# Comprehensive modeling of microRNA targets predicts functional non-conserved and non-canonical sites

**DOI:** 10.1186/gb-2010-11-8-r90

**Published:** 2010-08-27

**Authors:** Doron Betel, Anjali Koppal, Phaedra Agius, Chris Sander, Christina Leslie

**Affiliations:** 1 Computational Biology Program, Memorial Sloan-Kettering Cancer Center, 1275 York Avenue, New York, 10065, NY, USA; 2 Department of Computer Science, Columbia University, 1214 Amsterdam Avenue, New York, 10027, NY, USA

## Abstract

mirSVR is a new machine learning method for ranking microRNA target sites by a down-regulation score. The algorithm trains a regression model on sequence and contextual features extracted from miRanda-predicted target sites. In a large-scale evaluation, miRanda-mirSVR is competitive with other target prediction methods in identifying target genes and predicting the extent of their downregulation at the mRNA or protein levels. Importantly, the method identifies a significant number of experimentally determined non-canonical and non-conserved sites.

## Background

microRNAs are a class of small regulatory RNAs that are involved in post-transcriptional gene silencing. These small (approximately 22 nucleotide) single-strand RNAs guide a gene silencing complex to an mRNA by complementary base pairing, mostly at the 3' untranslated region (3' UTR). The association of the RNA-induced silencing complex (RISC) to the conjugate mRNA results in silencing the gene either by translational repression or by degradation of the mRNA [1]. Reliable microRNA target prediction is an important and still unsolved computational challenge, hampered both by insufficient knowledge of microRNA biology as well as the limited number of experimentally validated targets.

Early studies of target recognition revealed that near-perfect complementarity at the 5' end of the microRNA, the so-called "seed region" at positions 2 to 7, is a primary determinant of target specificity [2]. However, a perfect seed match by itself is a poor predictor for microRNA regulation due to the large number of random occurrences of any given hexamer in 3' UTRs.

Conversely, a number of studies have shown that some target sites with a mismatch or a G:U wobble in the seed region confer a noticeable regulatory effect [3-5], and a recent study using a cross-linking and immunoprecipitation (CLIP) method to study *in vivo *microRNA targets found a significant number of non-canonical sites [6,7]. Therefore, perfect seed complementarity is neither necessary nor sufficient for microRNA regulation.

Most computational methods require sites to have perfect seed complementarity ("canonical" sites) [8-10], with only a few methods allowing for G:U wobbles or mismatches in the seed region [11,12] ("non-canonical" sites). Other approaches consider predicted mRNA secondary structure and require energetically favorable hybridization between microRNA and target mRNA [13-15]. However, for the most part, all these target prediction methods generate a large number of predictions, many of which are presumed to be false. To address this problem, virtually all computational methods filter predictions by conservation, which eliminates poorly conserved candidate sites from consideration.

Several studies have used genome-wide mRNA expression changes following microRNA transfection to elucidate microRNA target specificity rules [8,9,16]. Grimson *et al. *defined a four-class hierarchy of canonical seed types of differing efficiencies and identified additional "context" features of target sites that correlate (but only weakly) with reduced expression levels, in particular the AU content flanking the target site. Using univariate regression between feature scores and expression change, they developed a seed-class-dependent scoring system called "context score", which has been incorporated into the TargetScan prediction program. Nielsen *et al. *assessed the significance of similar features by the shift in the cumulative distribution of log expression ratios using the same four-class seed hierarchy. Recently, proteomics studies of protein expression changes in response to microRNA transfection and knockdown [17,18] corroborated a number of these specificity features. Importantly, these studies showed that most targets with significantly reduced protein levels also experienced detectable reduction in mRNA levels, indicating that changes in mRNA expression are reasonable indicators for microRNA regulation.

Here we present a new algorithm called mirSVR for scoring and ranking the efficiency of miRanda-predicted microRNA target sites by using supervised learning on mRNA expression changes following microRNA transfections. mirSVR incorporates target site information and contextual features into a single integrated model, without the need to define seed subclasses. We use support vector regression (SVR) to train on a wide range of features, including secondary structure accessibility of the site and conservation.

We first compared mirSVR against a number of existing target prediction algorithms using a large panel of independent microRNA transfection and inhibition experiments as test data. For a fair comparison, we limited consideration to sites with canonical seed pairing in this analysis. mirSVR performs as well as, and often better than, existing methods for the task of predicting the extent of downregulation of genes at the mRNA or protein level. The miRanda-mirSVR approach effectively broadens target prediction beyond the standard notion of seed hierarchy and strict conservation without introducing a large number of spurious predictions. In particular, we found that the mirSVR scoring model correctly identified functional but poorly conserved target sites, and that imposing a conversation filter results in a reduced rate of detection of true targets.

mirSVR downregulation scores are calibrated to correlate linearly with the extent of downregulation and therefore enable accurate scoring of genes with multiple target sites by simple addition of the individual target scores. Furthermore, the scores can be interpreted as an empirical probability of downregulation, which provides a meaningful guide for selecting a score cutoff. We found that the model can correctly identify genes that are regulated by multiple endogenous microRNAs - rather than transfected microRNAs whose concentrations are above physiological levels - by analyzing targets bound to human Argonaute (AGO) proteins as identified by AGO immunoprecipitation [19]. We also revisited the idea of the seed hierarchy, and found that different seed types had wide and overlapping ranges of efficiencies. Finally, we tested the usefulness of including non-canonical sites in the model by evaluating performance on biochemically determined sites from recent Photo Activatable Ribonucleoside enhanced CLIP experiments (PAR-CLIP). In this data set approximately 7% of the detected sites do not contain perfect microRNA seed match to the expressed microRNAs [7]. We found that miRanda-mirSVR indeed correctly identified a significant number of these experimentally verified non-canonical sites. miRanda target sites and mirSVR scores are available at http://www.microRNA.org.

## Results and discussion

### mirSVR performance: efficiency of canonical sites and the role of conservation

#### Training the mirSVR scoring model

**Figure 1:**
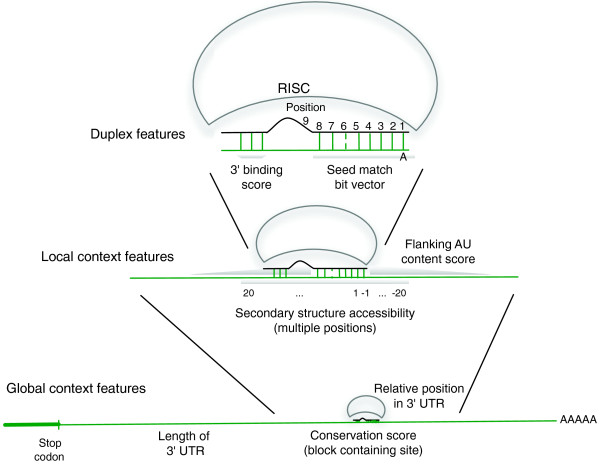
**Features used in the mirSVR model**. mirSVR uses features derived from the miRanda-predicted miRNA::site duplex, the local context of the candidate site, and the global context of the site in the 3' UTR. Duplex features include a bit representation of base-pairing at the seed region and the extent of 3' binding. Local features include AU composition flanking the target site and secondary structure accessibility score. Global features include length of UTR, relative position of target site from UTR ends, and conservation level of the block containing the target site.

The mirSVR algorithm learns to predict target site efficiency by training on mRNA expression data from a panel of microRNA transfection experiments. Training examples consist of genes containing a single candidate target site for the transfected microRNA in the 3' UTR. Target sites are represented by a set of binary features of the predicted miRNA::site duplex as well as local and global contextual features (Figure 1), together with its output label, given by the log expression change after microRNA transfection. The local contextual features include the AU content flanking the target site and predicted secondary structure accessibility at positions flanking the site, while global contextual features include the relative position in the 3' UTR, UTR length, and conservation (see Methods). Different seed types, including non-canonical sites, are therefore represented in a unified manner, and conservation is used as a feature rather than a filter. mirSVR learns the features weights using the support vector regression (SVR) algorithm, a variant of the well-known SVM algorithm [20] that uses real-valued outputs rather than discrete class labels.

For all results reported below, we trained mirSVR on a set of nine microRNA transfection experiments performed on HeLa cells from Grimson *et al. *[8]. We evaluated two different training modes for our model: (1) training only on genes containing a single canonical site in the 3' UTR, called the "canonical-only" model; (2) training on genes containing a single canonical or non-canonical site in the 3' UTR, where we allow non-canonical sites with exactly one G:U wobble or mismatch in the 6-mer seed region, called the "all-sites" model. The first mode produces a model that is readily compared with most existing target prediction methods, which largely assume at least a 6-mer seed match, while the second mode allows us to assess whether we can achieve statistically significant prediction results on non-canonical sites. Consistent with previous studies [8,9], the most significant features are base-pairings at the seed region and the sequence composition flanking to the seed region (Additional file 1, Figure S1). Additional features such as conservation, position in the UTR, and UTR length are weakly correlated with the extent of downregulation.

#### mirSVR scores improve ranking of canonical sites over existing target prediction methods

We first tested the canonical-only mirSVR prediction model, where we restricted consideration to genes with single canonical target sites, that is, sites with perfect complementarity to positions 2 to 7 of the microRNA. The test data consists of 17 independent microRNA transfection experiments followed by mRNA expression profiling from Linsley *et al. *[21], five microRNA transfection experiments followed by protein expression measurements from Selbach *et al. *[17], and three microRNA inhibition experiments followed by mRNA expression profiling [21-23].

We compared the performance of the mirSVR model against well-known existing target prediction methods that were representative of the different methodologies, namely: TargetScan's context score [8], which incorporates contextual feature scores estimated from expression data from transfection experiments and, like mirSVR, was optimized to predict the expression changes of the target genes; miRanda's alignment score [11,24], which was designed to score the quality of the miRNA::site duplex using dynamic programming and was the first method to incorporate binding at the 3' end of the microRNA; and PITA's energy score [15], derived from a secondary structure based method which computes the difference between the free energy of the predicted microRNA-target duplex and the energetic cost of unpairing the local secondary structure of the target site. For a general performance measure, we computed the Spearman rank correlation between the observed log expression change and the prediction score, which gives a general measure of the overall *ranking *performance of the algorithm. It is important to note that for this analysis, we did not filter the potential canonical target sites for conservation: mirSVR and comparison methods were required to rank all sites with seed matches, whether or not the sites are conserved. In this sense, we are not performing a typical method comparison of existing target prediction programs as they are implemented through various web servers. Instead, we are assessing the intrinsic value of different target site scoring systems to predict the extent of microRNA regulation.

**Figure 2:**
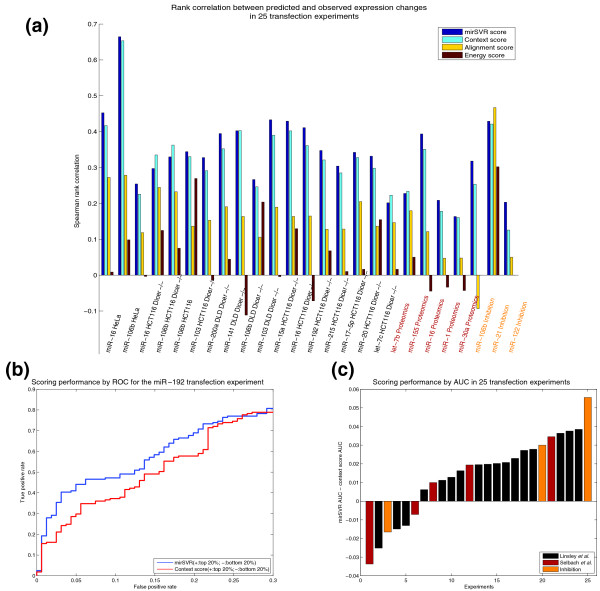
**Comparison of mirSVR to other methods**. **(a) **Spearman rank correlation (vertical bars) between prediction and observation for canonical seed targets as ranked by mirSVR score, context score, alignment score from miRanda and energy score from PITA. Rank correlations were computed between prediction scores and observed log expression changes for 17 test sets measuring mRNA expression changes following microRNA transfection in different cell lines and genetic backgrounds [21] (brown), five test sets measuring protein expression changes following microRNA transfection [17] (red), and three test sets measuring mRNA expression changes following microRNA inhibition [21,23,41] (orange). Ranking by mirSVR scores outperforms that by context scores in 21 out of the 25 test sets. **(b) **ROC curves (receiver operating characteristic) for mirSVR score versus context score for ranking the top 20% most downregulated targets (defined as true positives) and 20% of least downregulated targets (defined as true negatives) for the miR-192 transfection [21]. Shown here are the ROC curves up to 30% false positive detection. In this example, in the range shown, for a given false positive rate, mirSVR ranking yields an advantage of up to 10 percentage points in the rate of true positive prediction. **(c) **A summary of this ROC analysis over the 25 test sets, computing the area under the ROC curve (AUC) for mirSVR and context score and reporting the difference in performance (mirSVR AUC - context score AUC) for each test set. Overall, mirSVR score shows a statistically significant improvement over context score with a mean AUC of 0.80 as compared to 0.78 and outperforming context score in 19 (bars above the zero line) out of the 25 test sets (*P*-value < 0.006, signed rank test).

Our results show that when trained on canonical seed sites and using our full feature set, mirSVR strongly outperforms the alignment-based (miRanda) and energy-based (PITA) scores for the task of ranking single-site genes by their downregulation (upregulation) in response to microRNA transfection (inhibition), as shown in Figure 2a. We note that the miRanda and PITA alignment scoring systems were not trained on genome-wide expression data and in particular were not optimized for the task of ranking expression changes, as assessed here. Therefore, we would not expect these methods to perform as well as supervised approaches such as mirSVR. The context score method is the only other approach in our main comparison that exploits training data from microRNA transfection experiments. mirSVR performs better than context score in 21 out of the 25 test sets, which constitutes a statistically significant improvement (*P *< 0.002, signed rank test). The inclusion of a conservation measure into the mirSVR model does not account for the entire performance gain. After removing the conservation feature, mirSVR still outperforms context score in 18 out of the 25 test cases, suggesting that the learning algorithm - not just the inclusion of additional features - contributes to the performance gain.

In addition to the Spearman rank correlation, we compared the performance of mirSVR and context score by an ROC analysis where the true positive and true negative sets are defined as the top and bottom 20% of candidate target genes based on their expression changes following microRNA transfection (or inhibition) (Figure 2b). Consistent with the rank correlation results, mirSVR has a larger AUC (area under the ROC curve) than context score in 19 out of the 25 test cases (*P *< 0.006, Figure 2c). The results from both the rank correlation and ROC analysis indicate that mirSVR improves target ranking over the context score method for both reduction of mRNA levels and reduction of protein levels.

We also did a more limited comparison of mirSVR against context score, miRanda, PITA and two additional methods for which we could obtain published target site predictions but had no access to source code: PicTar [10] and Diana-microT [25]. In contrast to our main method comparison (Figure 2), here we were restricted to a limited number of target sites that were predicted by both additional algorithms, and in particular all sites were required to pass the conservation filter imposed by PicTar. For statistically meaningful results, we considered only experiments for which ≥ 50 targets were scored by all methods. Even when limited to a small set of conserved targets, mirSVR improves over all other methods in 8 out of 11 experiments in the Linsley *et al. *data set when evaluated in terms of rank correlation with extent of downregulation (Additional file 1, Figure S2a); for the other test sets, no experiments contained enough scored targets to make a comparison. Moreover, when assessing the mean log expression change of the top 50 predictions of each method, mirSVR's top predictions exhibit greater downregulation than those of any other method (Additional file 1, Figure S2b).

#### mirSVR detects genes with effective but non-conserved sites

**Figure 3:**
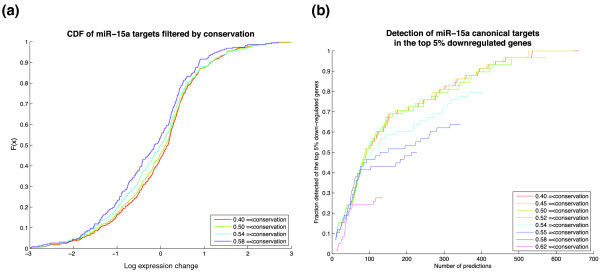
**Role of conservation in target prediction**. **(a) **Empirical cumulative distribution of log expression changes of genes with single canonical sites for miR-15a, filtered by increasing conservation thresholds. Distributions of more conserved sites display a subtle shift towards negative values indicating a slight increase in downregulation of target genes. **(b) **Detection rate of miR-15a targets defined as genes with a single canonical miR-15a site that are in the top 5% most downregulated genes (443 genes). Under increasing conservation thresholds, the detection rate of the most downregulated miR-15a targets drops substantially, showing loss of detection of genes with effective but non-conserved sites. Detection rates were scaled by the maximum number of miR-15a targets identified in the top 5% most downregulated genes without conservation filtering (red line).

Previous reports have shown that the most downregulated microRNA targets in transfection experiments are enriched for conserved target sites and more generally that target site conservation correlates with the extent of downregulation [8,9,26]. Many target prediction methods therefore use a conservation filter to remove what are assumed to be spurious predictions. We also found that increased conservation of the target site is correlated with increased suppression of the target genes by observing (i) a downward shift in the cumulative distribution of the log expression changes of more conserved targets (Figure 3a) and (ii) a negative weight for the conservation feature in the mirSVR model (Additional file 1, Figure S1).

However, for the task of detecting the most downregulated targets with single canonical sites in the Linsley *et al. *and Selbach *et al. *test sets, we found that the *detection rate *as a function of the number of predictions did not improve at any point by imposing a more stringent conservation filter (Figure 3b). If it were a good idea to filter mirSVR results for conservation, we would expect to see the detection curve for more conserved sites to climb more steeply than the detection curve for less conserved sites; instead, the detection curves for conservation filters all initially climb at the same rate. Eventually, as we run out of conserved sites that are in the 5% most downregulated set, the more conserved detection curves plateau at a lower detection rate, showing that a substantial number of downregulated targets are missed. We note that this effect is not restricted to our particular choice of conservation measure or even to the mirSVR scoring system. We repeated the analysis with context scores downloaded from TargetScan and using their associated conservation scores (*P_CT _*) [26] and similarly found no improvement in detection rates of the most downregulated targets with increased *P_CT _*threshold (Additional file 1, Figure S3). These results, which are consistent with previous work [14], suggest that conservation should be used in combination with other informative features to score target sites and not as hard filter, which leads to a substantial loss of *bona fide *targets.

### A unified scoring model for microRNA target sites

#### Interpreting mirSVR scores in terms of downregulation

The analysis so far has focused on genes with single canonical microRNA target sites for a straightforward comparison to existing methods. To obtain a unified model for a wider range of sites, we retrained mirSVR on all genes in the Grimson *et al. *data set containing either a single canonical target site or a single non-canonical site with at most a single G:U wobble or mismatch in the seed region. We confirmed that the "all-sites" mirSVR model performed similarly to our "canonical-only" mirSVR model for the task of predicting downregulation of canonical target genes (Additional file 1, Figure S4).

**Figure 4:**
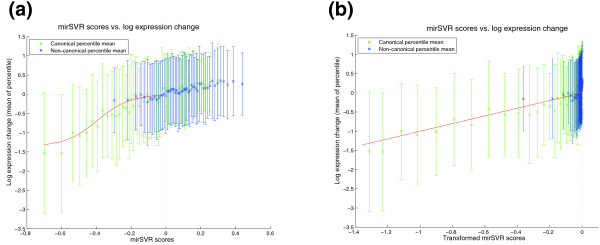
**Correlation of mirSVR scores with log expression change for genes with single canonical (green) and non-canonical sites (blue)**. mirSVR scores are divided into equal size bins (percentile) and the mean and standard deviation of the corresponding log expression changes are plotted for each bin. **(a) **Before sigmoid transformation, the mirSVR scores have non-linear correlation with the mean (*Z*-transformed) observed log expression change of the genes. Canonical target sites are generally more effective sites than non-canonical sites as shown by their more negative mirSVR scores and corresponding log expression change. Where scores for non-canonical sites fall in the same range as canonical sites, the corresponding mean expression change also fall in the same range, indicating that non-canonical and canonical sites with comparable scores inhibit their targets with similar efficiency. **(b) **After transforming with a sigmoid transfer function (fitted on the training data), mirSVR scores correlate linearly with log expression change and therefore can be used for analysis of target site efficiency; moreover, transformed site scores can be added to score genes with multiple sites.

**Figure 5:**
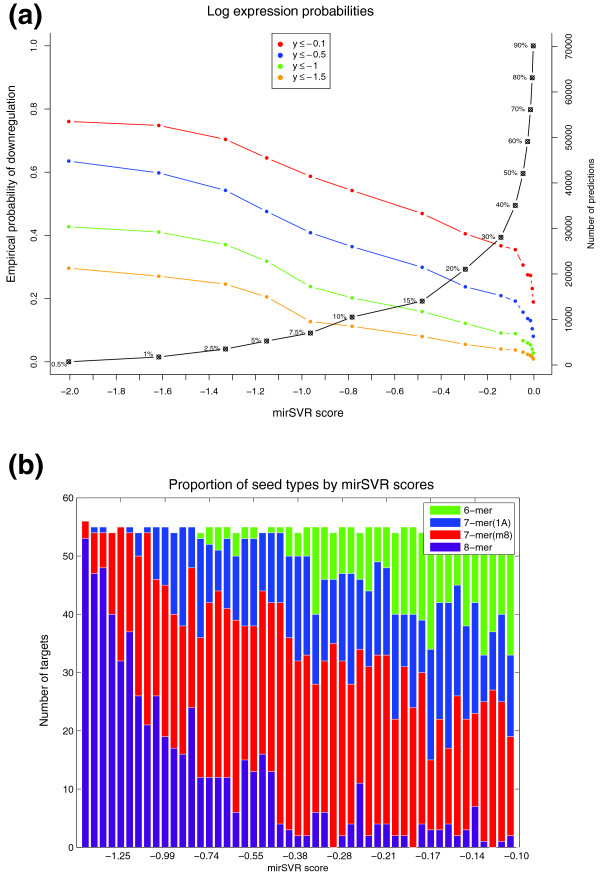
**Probability of downregulation and seed class distributions derived from mirSVR score analysis**. **(a) **Empirical probabilities of microRNA-mediated downregulation for different mirSVR scores. Using mirSVR prediction scores on the Linsley *et al. *data, we compute the empirical probability that a gene's *Z*-transformed log expression change is below *a *(*a *= -0.1, -0.5, -1.0, -1.5), conditioned that its (sigmoid-transformed) mirSVR score is less than a threshold *S *(*x*-axis). Points on the plot represent mirSVR score cutoffs *S *and their corresponding probability *P*(*y *≤ *a*|*x *≤ *S*). The black curve represents the fraction of predictions with scores equal to or less than the cutoff scores. For example, 10% of predicted targets have a score of ≤ -0.8 and their expected probability of observing a log expression change of ≤ -0.5 is approximately 40%. **(b) **The proportion of the four seed classes: 8-mers, 7m8, 7A1 and 6-mer in equal-size mirSVR score bins. The canonical sites from Linsley *et al. *were divided into equal size bins and the proportion of the four seed classes is shown by color. As expected the score distribution correlates with seed type hierarchy (for example, 8-mers have generally more negative mirSVR scores than 7m8 sites). However, inspection of the top 30% predicted target sites (mirSVR score ≤ -0.1) highlights the broad overlapping distributions of the four seed types, suggesting that the classification of target sites to seed classes is inadequate to represent their relative efficiency.

We then scored genes in the test data with either single canonical or non-canonical sites and assessed the correspondence between mirSVR scores and observed log expression changes over mirSVR score percentiles. The correlation between the mirSVR scores and the observed log expression change is non-linear (Figure 4a): a small improvement in score corresponds to a large increase in actual inhibition near the top of the mirSVR score range but little change near the bottom of the score range. This non-linearity is problematic for modeling genes with multiple candidate sites: in order to score multi-site genes by summing target site scores, individual site scores must contribute additively to target inhibition, which will only hold if individual scores correlate linearly with downregulation (Additional file 1, Figure S5). To correct for this effect, we fit a sigmoid transfer function between mirSVR scores and observed log expression changes (see Methods) that results in transformed scores that are linearly correlated with log expression change on both training and test data (Figure 4b) and thus can serve as a proxy for the extent of target downregulation. To better understand the correspondence between mirSVR scores and the efficiency of downregulation, we used the Linsley data set to estimate a gene's *empirical probability of downregulation*, which provides an estimate of the amount of downregulation given a mirSVR score. More precisely, for a given (*Z*-transformed) log expression reduction *a *< 0 and mirSVR score threshold *S*, we compute the empirical probability that a gene's expression change *y *is below or equal to *a *given that its score *f*(x) is smaller than or equal to *S *(Figure 5a). For example, genes that have a score of -1.0 or lower, corresponding to the top 7% of predictions, have more than a 35% probability of having a (*Z*-transformed) log expression change of at least -1 (downregulation by at least a standard deviation in terms of log expression changes) and better than 50% probability of a log expression change of at least -0.5 (Figure 5a green and blue curves). Thus, mirSVR scores can be converted to a probability of downregulation, which can be used as guide for selecting a meaningful cutoff for reporting target sites. The empirical distributions suggest an intuitive score cutoff of -0.1 or lower, since for scores closer to zero the probability of meaningful downregulation drops while the number of predictions rises sharply.

#### Seed classes have broad ranges of efficiencies

Previous reports identified four seed types that roughly correlate with extent of downregulation (8 mer > 7(m8) > 7(A1) > 6 mer) [27]. After rescaling mirSVR scores to correlate linearly with downregulation, we reexamined the notion of seed hierarchy in terms of mirSVR scores. Consistent with previous observations, we found that the mean mirSVR score by seed type generally agreed with the reported class hierarchy, namely, that longer seed matches correlate with extent of downregulation. However, each seed type had a broad distribution of scores, with considerable overlap between the different seed types (Figure 5b). In particular, there is a large overlap between score ranges for 8-mer sites and the 7(m8) sites and only a subtle difference between the 7(A1) and 6-mer distributions. Therefore, the distinction between seed classes and the subsequent rules used to rank their efficiency do not correctly capture the range of regulatory effect, and the assumption that longer complementarity in the seed region gives stronger inhibition does not always hold. We propose that our score-based method, which is independent of seed classification, provides a more meaningful ranking of target sites efficiency.

### Predicting the targets of endogenous microRNAs

#### mirSVR correctly extends to genes regulated by multiple endogenous microRNAs

So far we have measured mirSVR performance using expression data from microRNA transfection experiments. However, overexpression of microRNAs by transfection experiments may lead to stronger or more widespread downregulation than observed under physiological conditions and also appears to perturb endogenous microRNA regulation in the cell by out-competing the endogenous microRNAs for the silencing machinery [28]. In addition, the majority of cells express multiple microRNAs at significant levels [29] and most 3' UTRs have multiple predicted target sites for different microRNAs. It is therefore likely that under physiological conditions many genes are subjected to concurrent regulation by multiple microRNAs, and several target prediction methods model regulation by multiple microRNA sites [10,25]. To test the performance of the mirSVR all-site model on more physiological relevant targets, we generated another test set from published microarray data from AGO IP experiments [19]. RNA extracted from AGO1-4 immunoprecipitation was analyzed on a microarray platform and compared to RNA extracted from the washed lysate. The endogenous microRNA targets are identified as the set of genes that are enriched in the AGO-IP relative to the cleared lysate and contained a predicted microRNA target site for the endogenously expressed microRNAs.

We included in our prediction set genes with target sites for any or all of the top six endogenously expressed microRNAs (miR-16, miR-19b, miR-30e-5p, miR-32, miR-20a, miR-21). An ROC analysis where the true sites are the 20% most AGO-IP enriched genes and false predictions are the top 20% most enriched in the washed lysate achieved an AUC of 0.72. Moreover, of the top 20% most enriched genes in the AGO-IP, mirSVR correctly detected approximately 85% of these genes as targets of one or more of the endogenous microRNAs using a gene-level mirSVR score threshold of -0.1. In addition, we compared the mirSVR canonical-only model to context score using this AGO IP test set. Similarly to the transfection experiments, we found that mirSVR improves over context score both when comparing the rank correlation of the prediction scores with the enrichment in the AGO IP and by ROC analysis (Additional file 1, Figure S6). Therefore, although mirSVR was trained on data from microRNA overexpression experiments, which may include non-physiological targets, it makes meaningful target predictions for endogenous microRNAs expressed at regular cellular concentrations.

#### mirSVR identifies functional non-canonical sites

A number of studies have shown that non-canonical sites can lead to downregulation of target genes [3,30-32], although it is unclear whether these examples represent a widespread pattern of microRNA regulation. Recent large-scale biochemical identification of mammalian microRNA targets have shown that approximately 7% of the target sites are non-canonical [6,7] confirming that non-canonical sites account for an appreciable part of microRNA-mediated silencing. The correlation between mirSVR scores and downregulation shows that while canonical sites are generally more effective than non-canonical sites, canonical and non-canonical sites with similar mirSVR scores exert a similar regulatory effect on genes (Figure 4a). However, we still need to assess whether inclusion of non-canonical sites improves detection of microRNA-regulated genes or simply increases the fraction of false predictions.

**Figure 6:**
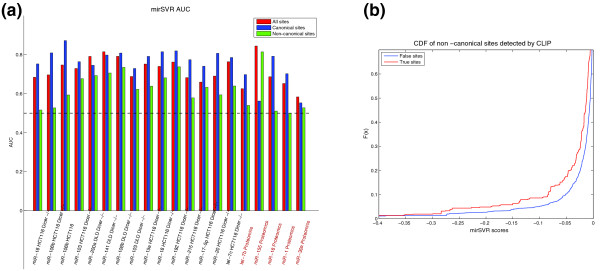
**mirSVR performance on non-canonical sites**. **(a) **A summary of the AUC scores for the Linsley *et al. *(brown) and Selbach *et al. *(orange) data sets. ROC analysis was performed on the most downregulated targets with log expression change of *Z*-score ≤ -1 (true positive) and the least regulated targets with *Z*-score ≥ 1 (true negative) for all sites, canonical sites only and non-canonical sites only. Note that two experiments were excluded due to low number of false positive and false negative examples. In all but one experiment the AUC values for non-canonical sites are above 0.5, indicating better than random detection. **(b) **A cumulative distribution function (CDF) plot of the mirSVR scores of the CLIP-identified non-canonical sites (true sites) and all other non-canonical sites predicted in the same 3' UTRs (false sites). The significant shift in the CDF for targets identified by the CLIP method indicates that mirSVR scores can identify a subset of the efficient non-canonical sites.

To investigate this question, we first performed an ROC analysis on the Linsley *et al. *and Selbach *et al. *test sets (inhibition data sets are too small for this analysis). In each of the transfection experiments we used the mirSVR all-site model to score three sets of predictions: i) only canonical targets, ii) only non-canonical targets and iii) all target sites. True positives for all sets are defined as targets with a log expression change (*Z*-score) ≤-1 and false predictions are targets with log expression change ≥ 1. The results show that when considering only non-canonical sites, the AUC values are significantly above random (average AUC 0.63, Figure 6a), indicating that mirSVR is able to discriminate between effective and ineffective non-canonical sites. Although the inclusion of non-canonical sites incurs some loss of performance, as measured by the average AUC for genes with only canonical sites versus all sites (AUC 0.76, 0.72 respectively), it enables detection of additional downregulated targets without greatly inflating false positives.

To further evaluate the performance of mirSVR on non-canonical sites, we used a new data set of biochemically verified microRNA target sites from PAR-CLIP experiments [7]. In this assay, the targeted mRNAs are covalently linked to AGO proteins and are identified by high-throughput sequencing after immunoprecipitation of the AGO protein. We focused the analysis on the approximately 7% of CLIP-identified sites that had no perfect 6-mer seed matches to any of the endogenous microRNAs, thus constituting a set of biochemically identified non-canonical sites. These sites were found both in coding regions and UTRs. To be consistent with how our model was trained, we further restricted the analysis to CLIP-identified non-canonical sites in the 3' UTRs that contained exactly one mismatch or G:U wobble in the 6-mer seed. We compared the mirSVR scores of the non-canonical candidate sites detected by CLIP (true sites) to those of non-canonical candidates in the same 3' UTRs that were not detected (false sites, see Methods). The distribution of mirSVR scores of the true non-canonical sites is shifted significantly downwards (indicating more confident predictions) relative to the false sites (*P *< 1.7e-36, one-sided KS test, Figure 6b). In addition, at a score cutoff of -0.1, mirSVR precision is 0.24 and the sensitivity is 0.09, significantly better than random prediction (*P *< 1.0e-4, Additional file 1, Figure S7), indicating that mirSVR scores are meaningful in discriminating non-canonical sites. However, the low sensitivity indicates that many of the functional non-canonical sites are not identified at this threshold. Future progress in identifying functional non-canonical sites is likely to require a more focused approach that includes training on additional experimental data.

Taken together, these results suggest that certain non-canonical sites are *bona fide *microRNA target sites that contribute, either in addition to canonical sites or independently, to gene silencing and that careful inclusion of such sites in the prediction model results in a more comprehensive target identification.

## Conclusions

We have presented a comprehensive microRNA target prediction and ranking algorithm that accurately predicts target site efficiency as measured by gene expression arrays, mass spectroscopy, enrichment in AGO-IP, and CLIP-based experiments. Evaluation by a variety of measures shows that miRanda-mirSVR is competitive with other methods when tested on mRNA and protein expression changes. We reexamined the use of conservation as a selection criteria for effective target sites to establish that site conservation is best used as a feature, not a filter. mirSVR scores are calibrated to correlate with downregulation and can be interpreted as an empirical probability of target inhibition, leading to an intuitive choice of score threshold. Finally, we have shown that non-canonical sites, as determined by the miRanda weighted alignment algorithm, can be judiciously included into the prediction method without inflating the number of false predictions, leading to detection of functional non-canonical sites as assessed on data from microRNA transfections and from CLIP experiments. mirSVR's improved performance can be attributed to a number of modeling choices and careful statistical analysis: using a representation that allows variability in seed region binding, including non-canonical seed base pairing; incorporating a wide range of microRNA::site duplex and contextual features; training with an algorithm that avoids overfitting; and correctly calibrating the contributions of individual sites in order to properly score multi-site targets. Our statistical analysis raises some questions regarding the common notion that extent of seed complementarity and conservation are primary determinants of functional sites and suggests that multiple features, some of which exert subtle effects, determine the efficacy of target sites.

### Future directions for microRNA target prediction

Although mirSVR scores incorporate many features important for microRNA-mediated inhibition, other potential aspects of target specificity are not included in the model. New data from high-throughput microRNA target identification experiments, such as cross-linking methods (HITS-CLIP [6], PAR-CLIP [7]) and Ago-IP pulldowns [19,33], reveals that, contrary to common belief, a significant portion of target sites are found in coding regions of mRNAs, which are not considered by most current target prediction methods. Predicting and scoring target sites in the coding region will likely require a specific model that accounts for features that are unique to these regions, such as polyribosome occupancy and translation rates. microRNA target specificity may vary substantially between organisms, given the diversity of RNAi pathways and the different constituents of RISC complexes. Moreover, it is entirely plausible that target specificity for a given microRNA could change substantially between different cell types. Likewise, additional non-specific sequence determinants that are currently unknown could influence microRNA-mediated regulation. For example, the inhibition of cog-1 by the nematode-specific lsy-6 microRNA is mediated by two target sites that are dependent on additional non-sequence-specific context features [4]. While it remains to be seen if such mechanisms are common, it is clear that one model may not account for all types of microRNA regulation. A number of RNA-binding proteins are known to be important post-transcriptional regulators that may have substantial effect on microRNA regulation, either through *cis *acting mechanisms, for example, by blocking target sites [34], or in *trans*, for example, by changing the secondary structure in the vicinity of the target site. In addition, a number of RNA-binding motifs have been linked to modulating microRNA-mediated regulation [35]. Finally, the balance between the abundance of microRNAs and of RISC is likely a critical determinant in microRNA-mediated regulation. Our recent study has shown that offsetting the balance between microRNA levels and RISCs by exogenous transfection of small RNAs can lead to a noticeable loss on endogenous microRNA regulations, presumably by out-competing the endogenous microRNAs for the limited RISC [28].

Therefore, integrating both microRNA and RISC expression levels into target prediction is an important goal towards more accurate modeling of microRNA regulation in a physiological context.

## Materials and methods

### Training and test data sets

#### Training data

The mRNA expression training data was taken from the Grimson *et al. *[8] [GEO:GSE8501] data set, containing expression arrays from HeLa cells transfected by miR-122a, miR-128a, miR-132, miR-133a, miR-142, miR-148b, miR-181a, miR-7, miR-9. Although mRNA expression was measured at 12 h and 24 h post-transfection, we used only the 24 h measurements since they gave stronger enrichment for downregulated targets with canonical seed matches (data not shown). Similar to the Grimson *et al. *study, we restricted our analysis to probes with signal intensities above median in the control transfection experiments. This filter is motivated by the fact that genes must be endogenously expressed at a reasonable level in order to be able to observe microRNA-induced silencing; moreover, this reduces the number of genes whose expression changes are induced by the introduction of the transfection vector. For training the mirSVR model, we included only genes that contained a single target site for the transfected microRNA, allowing only canonical sites for the canonical-only model and including restricted kinds of non-canonical sites for the all-sites model, as described below. We did not exclude single-site genes whose log expression change after transfection was positive.

#### Test data of microRNA transfection with mRNA expression measurements

The mRNA expression test data set was taken from the Linsley *et al. *study [21] [GEO:GSE6838], which comprised expression data from let-7c, miR-103, miR-106b, miR-141, miR-15a, miR-16, miR-17-5p, miR-192, miR-20, miR-200a, and miR-215 transfection experiments (all measured after 24 h), and was processed in a similar fashion to training set.

#### Test data of microRNA transfection with proteomics expression measurements

Protein expression test data set consisting of let-7b, miR-155, miR-16, miR-1, and miR-30a transfection experiments was taken from the Selbach *et al. *study [17]. Protein expression changes were computed as the median of the log 2 expression changes of its measured peptides between transfection and control experiments. Only proteins with unique peptide count ≥ 10 were used to ensure unique protein identification.

#### Test data of microRNA inhibition with mRNA expression measurements

Inhibition test sets were collected from three sources: the first is miR-106b 2'-O-methyl inhibition from [GEO:GSM155605] [21], the second is A172 glioma cells treated with anti-miR-21 [GEO:GSM298113] [23] and the third is LNA inhibition of miR-122 [22], where expression levels from multiple probes per genes were averaged.

#### AGO IP test data

To generate a test set of mRNA targets for endogenously expressed microRNAs, we used a recent study by Landthaler *et al. *[19] that identified the mRNA profiles of immunoprecipitates (IP) of the four AGO1-4 proteins in HEK293 cells. To identify the genes associated with FLAG/HA-tagged AGO proteins complexes, the transcripts isolated from the IPs were analyzed by microarrays and compared to the mRNA from the cleared lysate. The gene set that is enriched in the IP samples in comparison the lysate defines the complement of gene targets of the endogenously expressed microRNAs. The microRNA profile in HEK293 cells includes a number of microRNAs which can be grouped by their seed sequence similarity. To generate the test set, we selected the most abundant microRNAs from the six most common seed families (hsa-miR-16, hsa-miR-30e-5p, hsa-miR-19b, hsa-miR-32, hsa-miR-20a and hsa-miR-21) and searched for their target sites in genes that are enriched in the AGO1-4 IPs. Microarray data from the IP experiments was downloaded from [36] and normalized using the *GCRMA *R package; log enrichment values were computed using the *limma *package.

#### CLIP data

Data was provided by private communication from the authors. Non-canonical sites were defined as sequence traces determined by the CLIP method that did not contain perfect seed matches to any of the top 100 endogenous microRNAs expressed in HEK293 cells. Sequence traces that matched coding regions or 5' UTRs were discarded and those that matched 3' UTRs were used to predict non-canonical target sites; in the latter case, the endogenously expressed microRNA with closest seed to the sequence trace was assigned to the site. Within each identified 3' UTR, non-canonical sites that overlap with the CLIP-bound sequences were considered as true sites and all other non-canonical candidate sites for the same microRNAs were labeled as false predictions. This procedure generated a data set of 4,692 negative sites and 883 positive sites for 54 microRNAs.

### Predicting target sites

In order to search for canonical seed matches and restricted non-canonical sites and to obtain predicted miRNA::target duplexes, we used a modified version of the miRanda algorithm [11], miRanda 2.0, using a score cutoff (-sc) of 120, gap opening and gap extension (-go -ge) of -9 and -4 respectively. The modified version excludes the first 5' base and last two 3' bases of the microRNA from the alignment and allows for only a single G:U or mismatch in the seed region (positions 2 to 7). The algorithm computes an optimal sequence complementarity alignment between the microRNA and mRNA using a weighted dynamic programming approach where matches in the seed regions have higher position-specific weights, resulting in alignments that strongly favor 5' base-pairing. 3' UTR sequences were downloaded from UCSC genome browser, with the longest UTR chosen from afilternative isoforms. "Canonical target" sites are defined as sites that contain minimally a 6-mer perfect match at positions 2 to 7 of the microRNA.

### Target site features

Each target site is represented by a feature vector that encodes binary descriptors of the microRNA::mRNA duplex, extracted from miRanda alignment outputs, and additional contextual information such as the UTR length, AU composition, conservation and secondary structure accessibility scores. The predicted miRNA::site is represented by a seed bit vector denoting matches at positions 2 to 8 of the microRNA and presence of nucleotide 'A' across from position 1; an additional bit for a match at microRNA position 9; and a feature for binding at the 3' end of the microRNA. The seed bit vector represents different types of canonical seeds as well as non-canonical seeds in a uniform manner.

AU composition scores and 3' binding were computed as previously described [8]. Briefly, AU scores are defined as the sum of the adenosine or uridine bases over a window of 30 bases flanking the target sites, inversely weighted by their distance from the target site. 3' binding is defined as the number of perfect base pairs between positions 12 to 17.

Accessibility scores were computed using RNAplfold [37] with the following parameters: *w *= 80, *L *= 40 and *u *= 8 on a window of 160 bases around the target site. In practice, the accessibility scores for positions -20 to +20 averaged over a window of two bases were used for the SVR model. We used phastCons scores [38] for target site conservation, which measures the conservation of nucleotide positions across multiple vertebrates. The features and expression values (that is, log expression changes for training data and Linsley *et al. *test set, proteomics expression for Selbach *et al. *test set, and IP enrichment for Landthaler *et al. *test set) were *Z*-score transformed.

### Context score and PITA scores

Context scores values were computed using the source code downloaded from [39] that implements the regression model described in Grimson *et al. *2007. Briefly, context score is composed of three regression values, which are specific to each seed class, that model the correlation between the AU composition, 3'-binding and distance from the nearer end of the UTR with mRNA downregulation. Target sites are first classified into one of the four seed classes: 6-mer, T1A 7-mer, m8 7-mer and 8-mer; the context score is computed as the sum of the three regression values specific to the seed class. The computed context scores are highly correlated with the scores downloaded from TargetScan release 5.0 (0.96 average Pearson correlation, see Additional file 1, Table S1). PITA scores were computed with code downloaded from [40] using default parameters. Target sites that did not match the position of our miRanda predicted sites (up to three bases) were discarded.

### Support vector regression

We adopted a support vector regression (SVR) approach to model the degree of microRNA regulation given a set of numerical features representing the microRNA binding site and additional contextual information. SVR uses labeled training data {(**x***_i_*, *y_i_*)}_*i *= 1...*m *_to learn a linear function

f(x)=〈w,x〉+b

that estimates the real-valued output *y *for an example from its feature vector **x**. As in support vector machine, **w **is called the weight vector and *b *the bias term. In contrast to ordinary least squares regression, SVR uses an epsilon-insensitive loss function,

L(f(x),y)=max(0,|f(x)−y|−ϵ),

so that the optimization problem only penalizes examples whose outputs fall outside an "epsilon tube" around the prediction function; here ϵ is a parameter chosen prior to training. SVR training was performed using the libsvm package with the following parameters: -s 4 -t 0 -c 1.0e-1 -n 5e-1 (that is, *v *SVR with a linear kernel).

### Non-linear transformation of prediction scores

mirSVR scores were transformed using the sigmoid function $t_{a,b,c}(x)=\frac{-c}{1+\exp\left(a\cdot \left(-x\right)+b\right)}$. To learn the *a, b, c *parameters we performed five-fold cross-validation on the training data (single-site genes from Grimson *et al. *data set) and assembled the mirSVR prediction scores computed on each of the held-out sets. We then fit the parameters of the sigmoid function using MATLAB nlinfit function which performs a non-linear regression of mirSVR scores from the five-fold cross validation against their (*Z*-transformed) log expression changes. Finally, we retrained mirSVR on all the Grimson *et al. *data and transformed mirSVR prediction scores on test data using the sigmoid transfer function.

## Abbreviations

3'UTR: 3' untranslated region; AGO: Argonaute protein; AGO-IP: AGO immunoprecipitation; AUC: area under the curve; CLIP: cross-linking and immunoprecipitation; PAR-CLIP: Photo Activatable Ribonucleoside enhanced CLIP; RISC: RNA-induced silencing complex; ROC: receiver operating characteristic; SVM: support vector machine; SVR: support vector regression.

## Authors' contributions

DB, CS and CL conceived the project. DB designed and implemented the algorithm and performed the analysis. AK and PA contributed to the computational analysis. DB and CL wrote the paper. CL helped to develop the algorithmic approach and supervised the research. All authors read and approved the final manuscript.

## Supplementary Material

Additional file 1 **Supplementary material and figures**. Supplementary data and figures. Click here for file
