# Supplementary material for: Comprehensive modeling of microRNA targets predicts functional non-conserved and non-canonical sites
Source: Genome Biol. 2010 Aug 27;11(8):R90. doi: 10.1186/gb-2010-11-8-r90 (PMC2945792; doi:10.1186/gb-2010-11-8-r90)
Supplement: Additional file 1 — Supplementary material and figures. Supplementary data and figures. [file gb-2010-11-8-r90-S1.pdf]

Supplementary data: Comprehensive modeling of microRNA targets predicts functional non-conserved and non-canonical sites

Doron Betel[1], Anjali Koppal[2], Phaedra Agius[1], Chris Sander[1], Christina Leslie[1]
[1]Computational Biology Program, Sloan-Kettering Institute, New York, NY
[2]Department of Computer Science, Columbia University, New York, NY

Comparison of context score values

Context score values were computed by parsing the AU composition, 3'-binding and UTR position features from predicted microRNA::target site alignments. Seed specific scores were calculated using the regression parameters described in Grimson et al. Table S6 [8] using the source code downloaded from [39]. Due to differences in predicted miRNA::target alignments, which in our study were generated by miRanda, the context score values computed based on our predicted duplexes (mirSVR-CS) are not exactly the same as those calculated by TargetScan (TargetScan-CS), and this difference may have some impact on the performance assessment.

To assess the significance of the differences between our computed context scores to those provided by TargetScan5.0, we first calculated the Pearson correlation between the two context score values for the canonical sites in the Linsley data set [21]. We found a significant correlation between the two scores (Supplementary Table 1), and most of the difference can be attributed to variations in the 3'-binding scores. We also correlated the two scores with the observed log expression changes and found that mirSVR-CS values are in fact better correlated with the observed expression changes than the context scores provided by TargetScan. We conclude that differences in predicting the base-pairing in the miRNA::mRNA duplexes, and the resulting differences in context score values, did not adversely affect context score performance in our comparative analysis.

Seed class	mirSVR-CS vs. TargetScan CS (Pearson)	mirSVR-CS vs. Log exp-change (Spearman)	TargetScan-CS vs. Log exp-change (Spearman)
8-mer	0.92 (p <2.2e-16)	0.32	0.30
7-mer m8	0.99 (p <2.2e-16)	0.33	0.32
7-mer A1	0.97 (p <2.2e-16)	0.30	0.28

Table S 1: **Comparison between mirSVR-CS and TargetScan-CS.** Pearson correlation between the computed context scores in this study (mirSVR-CS) and the context scores downloaded from TargetScan5.0 (TargetScan-CS) for three seed classes (first column); the Spearman rank correlations between mirSVR-CS and log expression change (second column); and Spearman rank correlations between TargetScan-CS and log expression change (third column). Note that 6-mer sites are not included since only a few such sites are reported by TargetScan.

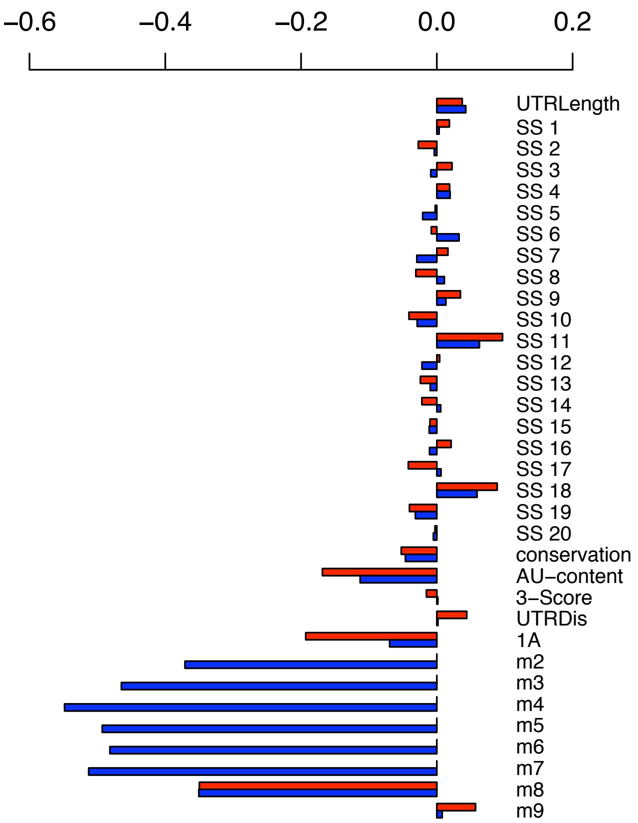

Figure S 1: **Regression coefficient values of mirSVR features.** The learned weights of the various features used in mirSVR canonical (red) and all-sites (blue) models. Negative values indicate that the feature contributes to down regulation of the target gene whereas positive values are disfavored. The features include: UTR length, secondary structure accessibility scores (SS) from position -20 to +20 averaged over a window of two; conservation [38]; AU-content around target site in a window of 30 bases upstream and downstream of the site; $3'$ binding score; relative distance from UTR end (UTRDis), and seed base pairing up to position 9 with additional bit to represent the presence of A across the first microRNA base (1A). In the canonical model (red bars), the coefficients of the seed region bit vector (m2-m7) are zero since all target sites are base-paired to the target mRNA between positions 2-7 and therefore do not contribute to the regression model.

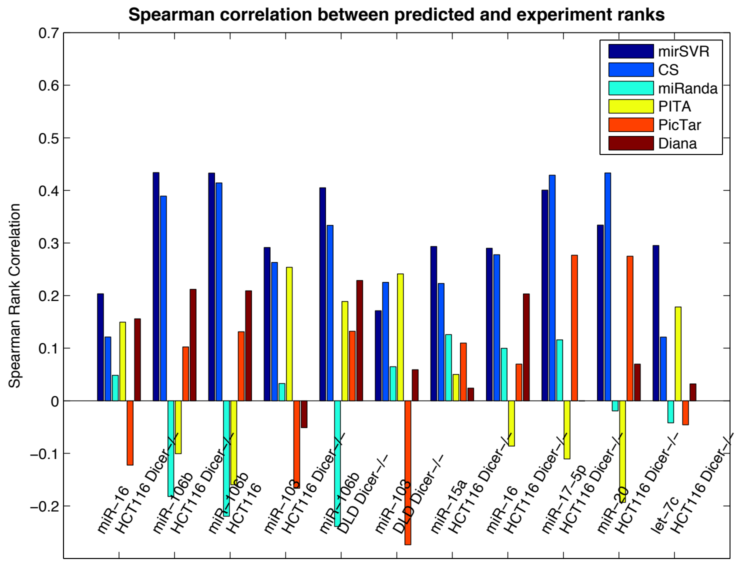

a

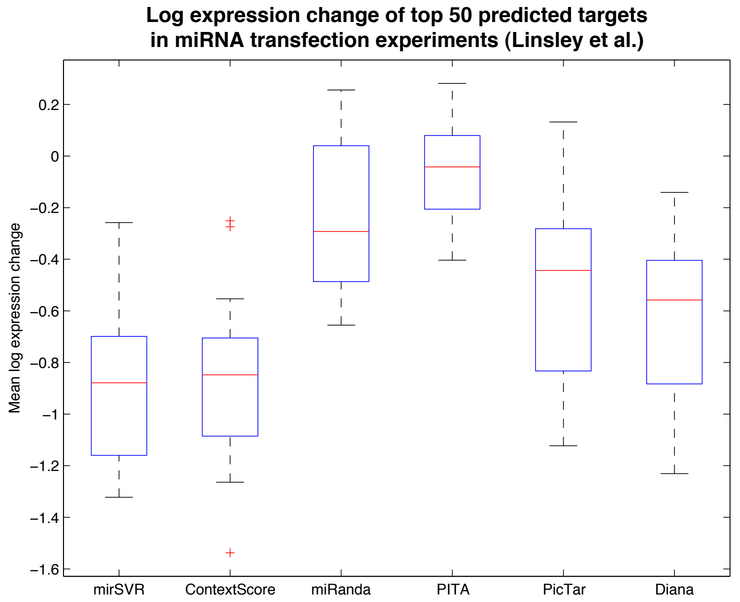

b

Figure S 2: **Comparison of target prediction methods.** (**a**) Spearman rank correlation between target site prediction scores and measured downregulation. Genes with single canonical sites were ordered by mirSVR, context score miRanda [11], PITA [15], PicTar (4way) [10], Diana-microT [25]. All methods were required to rank the same set of target sites, and the ranking of the sites from each method was compared the rank order of the Z-transformed log expression change. Due to the low number of predictions by PicTar for some of the Linsley et al. microRNA transfection experiments, not all experiments were included in the analysis. Overall, mirSVR outperforms all other methods in 8 out of 11 experiments, supporting our conclusion that mirSVR improves over the mostly commonly used target prediction methods. (**b**) The mean log expression change of the top 50 predicted targets from each prediction method were computed in each of the miRNA transfection experiments. The results are summarized in the box plots where red lines represent the median value over the 17 experiments, 50% of the scores are within the blue box, and 75% are within the dashed lines. Overall, the top predictions from mirSVR are more downregulated compared to the top predictions of any other method.

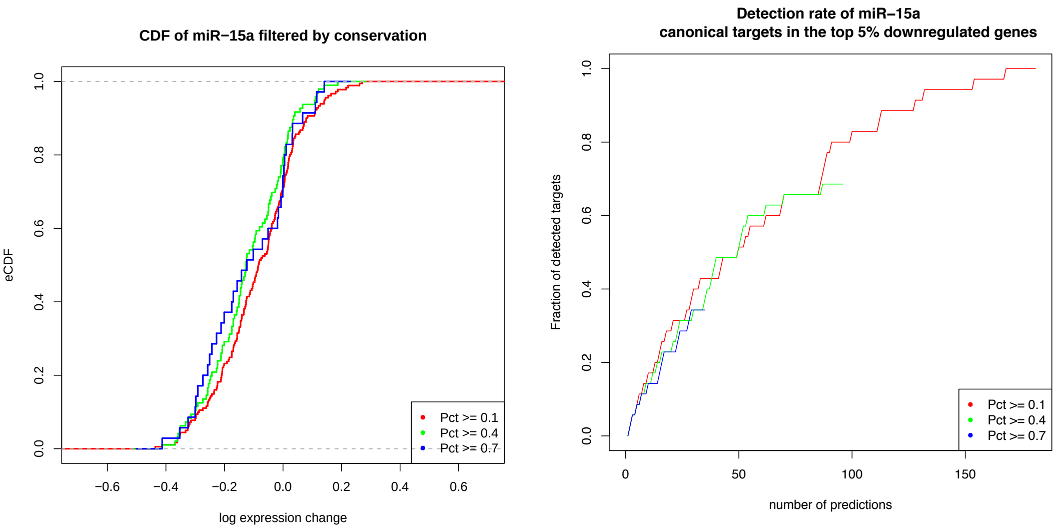

a **b**

Figure S 3: **Analysis of P_{CT} conservation in target prediction.** (**a**) Similarly to Figure 3a in the main text, we computed the distribution of TargetScan targets in the miR-15a transfection experiments under increasing P_{CT} threshold values. Consistent with previous reports and our own analysis, the CDF plots show a shift towards the negative values for the more conserved sites, indicating a correlation between conservation and extent of downregulation. (**b**) Detection rate of the top 5% most downregulated target genes in the miR-15a experiments. Similarly to Figure 3b in the main text, increasing the conservation threshold of miR-15a targets does not improve the detection rate of the most downregulated targets. Thus, relaxing the conservation threshold does not necessarily introduce false predictions as many of the most downregulated genes include poorly conserved sites. Detection rates were normalized by the maximum detection rate achieved by the lowest conservation value (i.e. red line).

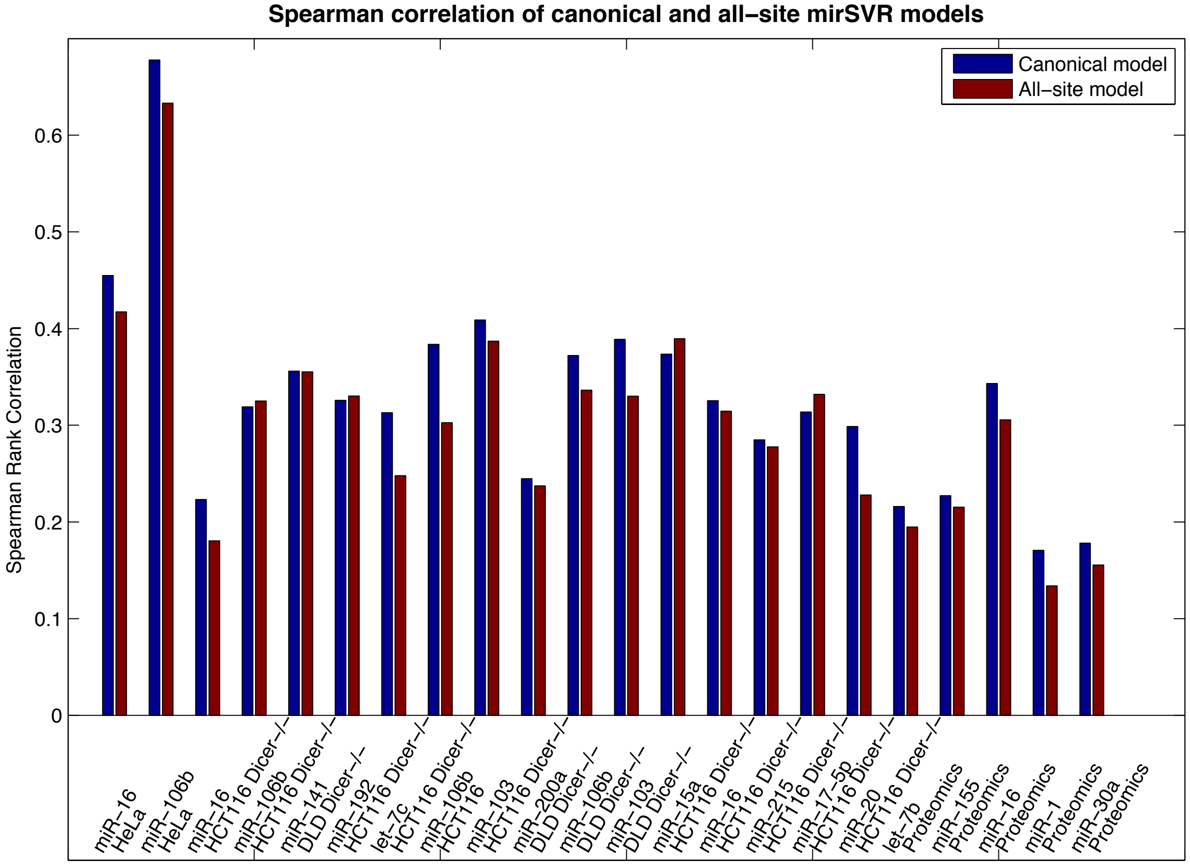

Figure S 4: **Comparison of mirSVR canonical and all-sites models.** The Spearman rank correlation of mirSVR scores for genes with single-canonical sites from the Linsley et al [21] and Selbach et al [17] (last 5 experiments labeled as proteomics) shows similar performance for both models with a slight advantage for the canonical model.

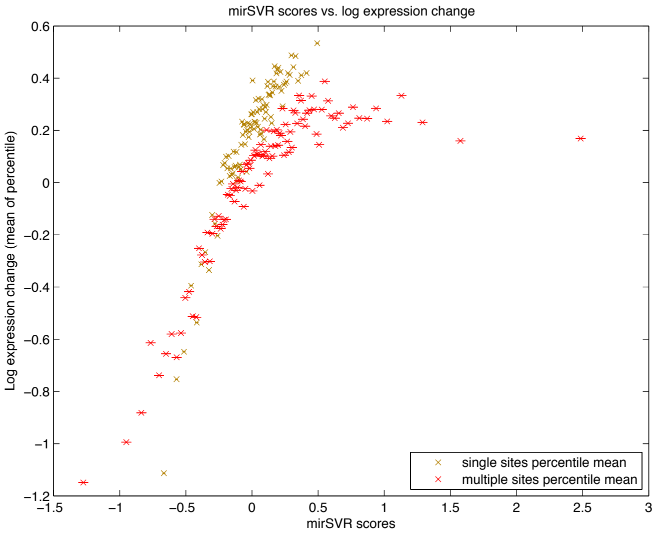

a

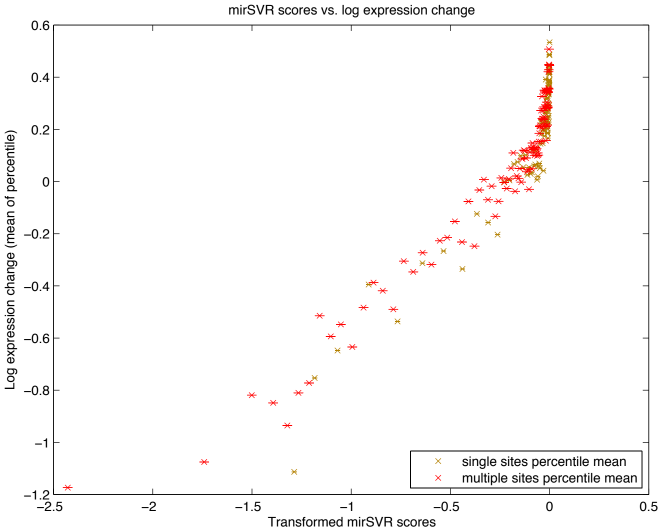

b

Figure S 5: **Non-linear transformation of mirSVR scores calibrates the scores to be linearly correlated with log expression changes.** (**a**) Due to the non-linear correlation of raw mirSVR scores with expression change, summing the scores of individual target sites in multi-site genes tends to overestimate downregulation. As a result, genes with single sites are not correctly ranked against genes with multiple sites, and the two sets appear as separate groups in the scatterplot of scores versus expression changes. (**b**) After non-linear transformation by a sigmoid function, the scores for single and multiple site genes are directly comparable and correctly interleaved in the score ranking, as shown in the scatterplot of transformed scores versus expression changes.

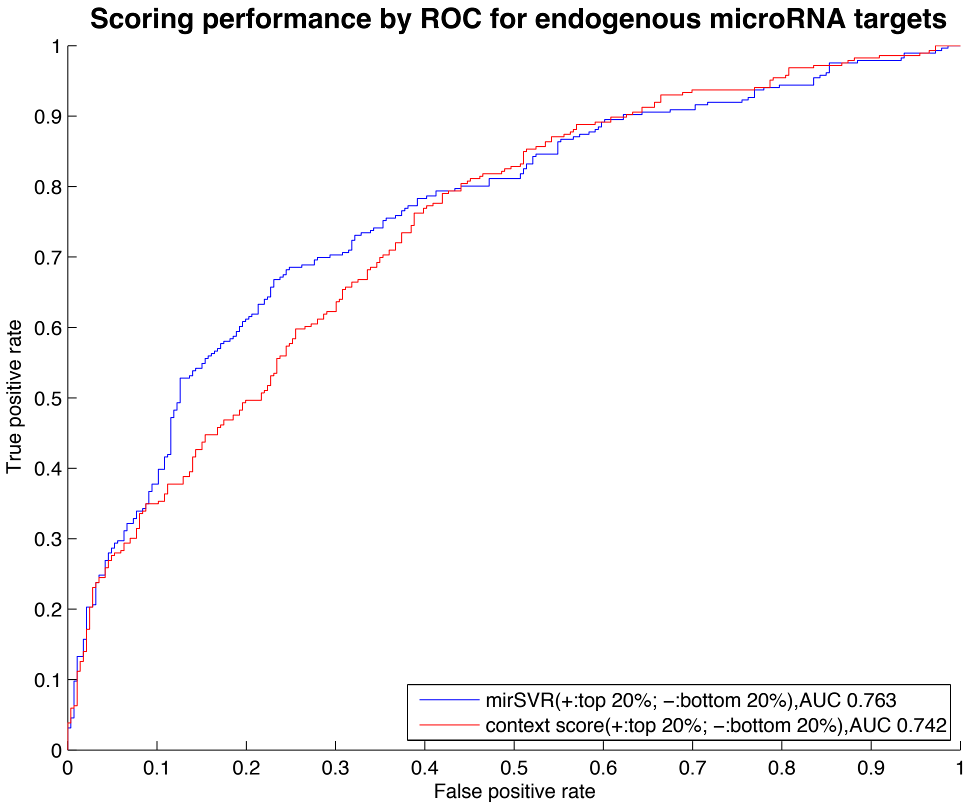

Figure S 6: **Comparison of mirSVR to context score on *in vivo* regulation by endogenous microRNAs.** To test the effect of multiple target sites on the inhibition of targets under physiological condition we analyzed the inhibition of targets of the top six microRNAs expressed in HEK293 cells as measured by AGO-IP enrichment [19]. We compared the performance of mirSVR canonical-only model to context score by Spearman rank correlation and ROC analysis. In the Spearman rank comparison of 1428 genes, mirSVR achieved a correlation of 0.35 whereas context score achieved a correlation of 0.33. Similarly, the AUCs are 0.763 and 0.742 for mirSVR and context score, respectively.

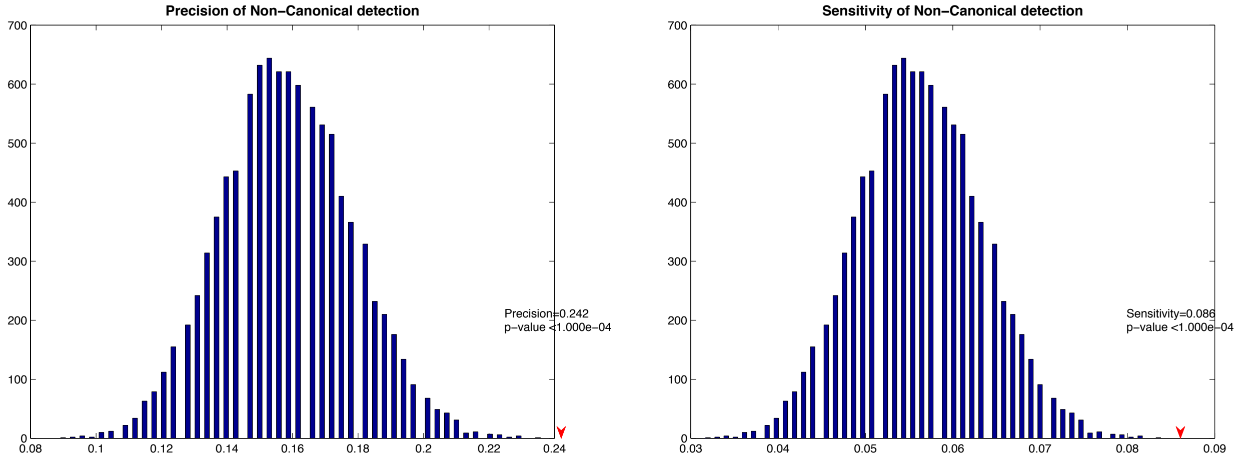

a **b**

Figure S 7: **Precision and sensitivity of mirSVR prediction of non-canonical sites.** (**a**) mirSVR precision, defined as TP/(TP+FP), was compared to a set of 10,000 randomized predictions, using a mirSVR score cutoff of -0.1 to define the number of predictions made. The precision attained by mirSVR (marked by red arrow) satisfies $p < 1.0\text{e-}4$ relative to the randomized predictions. (**b**) Similarly, mirSVR sensitivity, defined as TP/(TP+FN), satisfies $p < 1.0\text{e-}4$ relative to random predictions.
